# A Digital Fabrication of Dental Prosthesis for Preventing Self-Injurious Behavior Related to Autism Spectrum Disorder: A Case Report

**DOI:** 10.3390/ijerph18179249

**Published:** 2021-09-02

**Authors:** Seoung-Jin Hong, Yong Kwon Chae, Chunui Lee, Sung Chul Choi, Ok Hyung Nam

**Affiliations:** 1 Department of Prosthodontics, School of Dentistry, Kyung Hee University, Seoul 02447, Korea; ssabock@hanmail.net; 2 Department of Pediatric Dentistry, School of Dentistry, Kyung Hee University, Seoul 02447, Korea; pedochae@gmail.com (Y.K.C.); pedochoi@khu.ac.kr (S.C.C.); 3 Department of Oral and Maxillofacial Surgery, Wonju College of Medicine, Yonsei University, Wonju 26426, Korea; chunuilee@naver.com

**Keywords:** autism spectrum disorder, behavior guidance, digital dentistry, self-injurious behavior, special health care needs

## Abstract

This case report aimed to demonstrate the prosthetic solution of an autism patient with self-injurious behavior using digital dentistry. A 24-year-old male visited our clinic with chief complaints of severe gingival recession associated with self-injurious behavior. Bilateral fixed prosthesis with denture flange were delivered using a digital workflow for the protection of the gingiva. The patient showed healed gingival tissue, behavioral modification, and acceptable oral hygiene during the follow-up period. Also, his caregivers reported no recurrence of the self-injurious behavior. Autism patients usually show self-injurious behavior, which can damage their oral tissue. With adoption of this prosthesis, behavior modification as well as healing of oral tissue was achieved.

## 1. Introduction

Patients with autism spectrum disorder (ASD) present with unique behavioral impairment, characterized by poor communication and social interactions, as well as stereotyped behaviors and responses [1]. These features can compromise dental care and oral hygiene [2]. In addition, repeated self-injurious behavior (SIB) that causes severe oral tissue damage has been reported in ASD patients [3].

ASD patients may develop self-induced injuries in the head and neck region [4]. Any oral tissues can be involved including gingiva, mucosa, teeth, and tooth-supporting tissues. A previous study reported that tongues and lips were the predominantly affected sites in oral self-injuries [5]. A previous study regarding Chinese children with ASD reported that these patients showed parafunctional habits, such as biting hard objects (31.3%), bruxism (16.7%), and lip biting (9.7%) [6]. However, ASD patients are less sensitive to painful stimuli and less likely to express their physical discomfort; caregivers and clinicians may misinterpret that the patient is not in pain [7,8]. Therefore, without appropriate intervention, self-injured lesions can cause chronic inflammation, leading to severe damage of tissue.

The SIB can be managed by a combination of behavior modification therapy and physical restraints [9]. Physical restrictive devices such as arm splints, gloves, or bandages were used to protect the body parts, and application of dental protective appliances for the protection of the lip or tongue has been reported. In case of gingival injury, behavior modification therapy has been used to reduce SIB but was found ineffective [10]. However, studies regarding use of dental protective appliances for the protection of the gingiva have been limited.

This case report describes the prosthetic rehabilitation for protection against gingival injuries associated with ASD using digital dentistry.

## 2. Case Report

**Figure 1:**
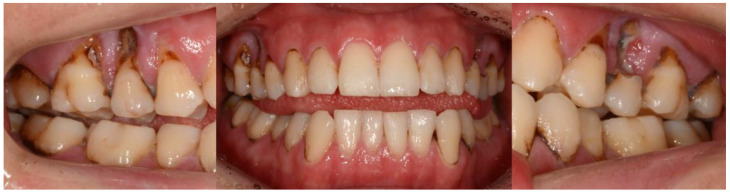
Gingival injury from self-injurious behavior.

**Figure 2:**
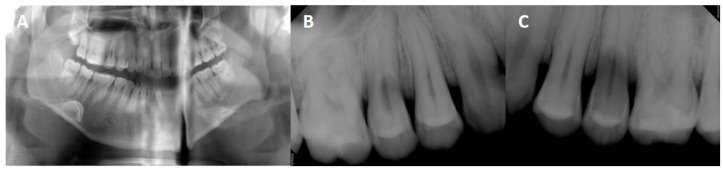
Pretreatment radiographs. (**A**), Panoramic radiograph. (**B**), Periapical radiograph of the maxillary right second premolar. (**C**), Periapical radiograph of the maxillary left second premolar. Severe dental caries with an extensive radiolucent area were observed in the root of the maxillary right and left second premolars.

A 24-year-old male visited our clinic, and the chief complaint was severe gingival recession. The patient had been diagnosed with ASD accompanying mental retardation and epileptic seizures. His caregivers reported SIB, with persistent finger scrapping in the maxillary posterior region. A clinical examination revealed severe buccal gingival recession of more than 5 mm with dental caries in the maxillary right and left second premolars and buccal gingival recession in the maxillary right and left first premolars, first molars, and second molars (Figure 1). Pretreatment panoramic radiograph and periapical radiographs of the maxillary right and left second premolars were taken. Precise imaging of radiograph images was challenging due to poor cooperation of patient; severe dental caries with extensive radiolucent area was observed in the root of the maxillary right and left second premolars (Figure 2). The maxillary right and left second premolars were planned to extract, because the loss of the root was extensive and the prognosis of the restoration of crown was poor.

The treatment plan was discussed with the prosthodontist, and the patient was offered a restrictive device: straight-arm splint or protective glove to control SIB. His caregivers refused the restrictive device treatment and opted for a restoration of bilateral fixed partial prosthesis with denture flange.

**Figure 3:**
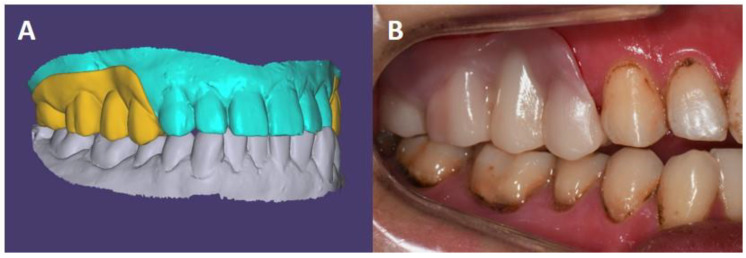
Fabrication of interim prosthesis. (**A**), The prosthesis was designed with CAD software (Exocad). (**B**), Clinical photo of the prosthesis. The margins of denture flange were adjusted during the follow-up period.

Under conscious sedation, preliminary impressions of both arches were taken. The interocclusal record was partially scanned in multiple areas, confirming the occlusion of posterior teeth by using an intraoral scanner (Medit i500; Medit Corp., Seoul, Korea). Diagnostic casts were scanned by using a desktop scanner (Medit T500; Medit Corp., Seoul, Korea), imported into a computer-aided design (CAD) software (Exocad; Exocad GmbH, Darmstadt, Germany), and aligned to the maximal intercuspal position using the scanned interocclusal record. Templates of interim restorations of the fixed partial denture with denture flange were designed (Figure 3). The interim restorations were fabricated by using a 3D printer (Raydent Studio 600; Ray Corp., Hwaseong-si, Korea) with photocurable resin (Raydent C&B; Ray Corp., Hwaseong-si, Korea) and pink acrylic resin (Tokuyama Rebase II; GC Corp., Tokyo, Japan), and preparation guides were also fabricated by using a 3D printer with photocurable transparent resin (Tera Harz TC-85DAC; Graphy Inc., Seoul, Korea). Under general anesthesia, the injured area was thoroughly examined, and the area to be protected was determined. The maxillary right and left second premolars were extracted, and the maxillary right and left first premolars, first molars, and second molars were prepared for zirconia-fixed partial dentures with denture flange under additional local anesthesia. The teeth were prepared anatomic occlusal reduction of 2 mm and axial reduction of 1 mm with a shoulder margin using a 1-mm tip round-end diamond bur (Shofu Inc., Kyoto, Japan). All line angles were rounded. The amount of tooth preparation and fitness of the denture flange of the interim restoration to the buccal gingiva were confirmed using the preparation guide. The interim restoration with denture flange was finally fabricated by using the 3D printed template and cemented with a zinc oxide eugenol cement (Temp-Bond^TM^; Kerr Corp., Orange, CA, USA). Daily use of a dental water jet and oral gargle was instructed to the patient’s caregivers. The operating time was about 2 h. In particular, the preparation procedure time was greatly reduced by using the preparation guide, and registering interocclusal relationship record procedure was not required; therefore, the overall operating time was reduced due to the digital device.

The status of the injured area and prosthetic complications were evaluated every month. Also, the border of the denture flange was adjusted to be thinner to prevent the hanging of fingers on the border.

**Figure 4:**
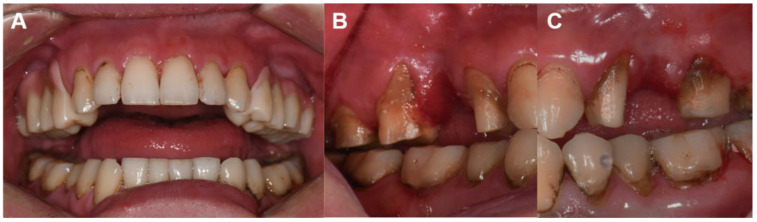
1-year follow-up data. (**A**), Definitive restorations of fixed prosthesis with denture flange. (**B**,**C**), Oral tissue and periodontal maintenance are clinically acceptable.

Under conscious sedation, the impression for the definitive restoration was taken using a custom tray with vinyl polysiloxane impression material (Imprint II Garant; 3M ESPE, St. Paul, MN, USA), and a definitive cast was fabricated and scanned by using a desktop scanner (Medit T500). The definitive restorations were designed on the CAD software, and the interim restorations were digitized and used as a reference to design the denture flange. The definitive restorations were milled by using a milling machine (DWX-52D; Roland, Hamamatsu, Japan) with a zirconia block (Natura Z; DMAX Corp., Seoul, Korea), and colored using coloring liquid (Aqua Base; DMAX Corp., Seoul, Korea) on the gingival area to keep the border of the denture flange thin. The definitive restorations were cemented with a zinc oxide eugenol cement (Temp-Bond^TM^), and daily use of a dental water jet and oral gargle was instructed. During the follow-up period, supportive periodontal cares were performed and the restorations were removed and re-cemented after cleaning every 3 months. One year after the placement of the definitive restoration, no complications or oral tissue injury were observed (Figure 4). Also, his caregivers reported no recurrence of the self-injurious behavior on the gingiva.

## 3. Discussion

High prevalence (approximately 42%) of SIB in ASD patients has been reported [11]. Due to its relatively high prevalence rate, clinicians should examine oral cavity of ASD patients thoroughly to find self-injury related lesions and provide an adequate intervention, if needed. A timely intervention with adequate intraoral appliance is critical for patients’ quality of life [12]. It enables patients with self-injurious behaviors to avoid preventable complications such as severe inflammation and irreversible tissue loss. A protective appliance should be designed with careful consideration of status of self-injured tissue, patient’s cooperation, and patient’s ability to maintain his oral hygiene [13]. Various properties of material such as strength, flexibility, and biocompatibility should also be considered [14].

In this case, SIB in an ASD patient resulted in damage to the gingival tissue, and the patient was restored with fixed prosthesis with denture flange using a digital workflow. Owing to this new technique [15], the fabrication procedure could be simplified and the operating time under anesthesia may be shortened. Duration of anesthesia exposure can determine the incidence of perioperative complications [16]. Additionally, as the practice of this case was based on digital dentistry, the patient data including intra-oral anatomical structures and design of prosthesis could be stored without any deformation or change. Therefore, the same appliance can be refabricated without further dental work such as impression-taking and additional visits to dental clinics in case of appliance loss or breakage. And this is beneficial especially to special healthcare needs patients who have limited access to dental clinics.

Typically, denture bases are fabricated with acrylic resin. Because of polymerization reaction, which is the nature of acrylic resin, dentures made with acrylic resin have a disadvantage in volumetric stability due to shrinkage, and this could cause undesirable volumetric change in denture fabrication and repair [17]. Besides, dentures made with acrylic resin have porous surfaces, and this could lead to hygienic problems during usage. On the other hand, volumetric change can be avoided when a denture is made with zirconia. It also has a lower affinity for bacterial plaque, and is suitable for a milling method in digital workflow [18].

In terms of behavioral modification, the appliance in this case not only protected involved gingiva but also successfully decreased gingiva-scraping frequency. However, it was reported that the appliance designed as a protector against self-injurious behavior may also cause development of alternative self-injurious behavior at other body parts [19]. Thus, guardians should be informed about this possible side effect and clinicians are responsible for additional intervention when a protective appliance is used in patients with self-injuries.

## 4. Conclusions

Autism patients usually show self-injurious behavior, which can lead to oral tissue damage. In case of self-injurious behavior in autism patients, a timely intervention is essential to provide better quality of life. Zirconia-fixed partial dentures with denture flanges were restored for prosthetic rehabilitation and protection against gingival injury from SIB in a patient with ASD. The digital workflow was advantageous for special healthcare needs patients who have limited access to dental clinics in terms of treatment procedure, time, and clinical outcome. The patient showed healed gingival tissue, behavioral modification, and acceptable oral hygiene.

## Data Availability

Data are available upon reasonable request.
